# Supplementary material for: Complexome profiling of the Chlamydomonas psb28 mutant reveals TEF5 as an early PSII assembly factor
Source: Plant Cell. 2025 Mar 17;37(6):koaf055. doi: 10.1093/plcell/koaf055 (PMC12164590; doi:10.1093/plcell/koaf055)
Supplement: koaf055_Supplementary_Data [file koaf055_supplementary_data.zip › psb28_interactive.zip!/plots/intensity/qualityPlots/peptides/_Cre16.g687200.t1.1.html]

### Cre16.g687200.t1.1

Trivial name:   
SeqCoverage [median]: 38.50  
Mass: 16.77 kDa  
  
 

| Sequence | MissedCleavage | Mass [Da] | wtIntensity | muIntensity | Score |
| --- | --- | --- | --- | --- | --- |
| AADLVPDSVPR | 0 | 1138.60 | 4969.18 | 4565.82 | 94.41 |
| AAKDDKDDMVSGVFK | 2 | 1624.78 | 417.39 | 0.00 | 40.80 |
| AGGGKGSDLDDPLAEAR | 1 | 1627.78 | 6755.64 | 3009.98 | 136.34 |
| DDDDAIDVTPK | 0 | 1202.53 | 0.00 | 410.21 | 134.68 |
| GSDLDDPLAEAR | 0 | 1257.58 | 2289.49 | 4684.57 | 190.86 |
| TASGLVDK | 0 | 789.42 | 2011.40 | 3917.53 | 94.11 |
| TASGLVDKAADLVPDSVPR | 1 | 1910.01 | 1633.37 | 2872.24 | 68.41 |
|  |  |  | 18076.5 | 19460.4 |  |
